# Pathogenomics Characterization of an Emerging Fungal Pathogen, *Fusarium oxysporum* f. sp. *lycopersici* in Greenhouse Tomato Production Systems

**DOI:** 10.3389/fmicb.2020.01995

**Published:** 2020-08-27

**Authors:** Tika B. Adhikari, Anne Gao, Thomas Ingram, Frank J. Louws

**Affiliations:** ^1^Department of Entomology and Plant Pathology, North Carolina State University, Raleigh, NC, United States; ^2^Department of Microbiology, Immunology and Molecular Genetics, University of California, Los Angeles, Los Angeles, CA, United States; ^3^Department of Horticultural Science, North Carolina State University, Raleigh, NC, United States

**Keywords:** tomato (*Solanum lycopersicum)*, Fusarium wilt, *Fusarium oxysporum* f. sp. *lycopersici*, virulence, host resistance, genetic diversity, fungal effectors, *SIX* genes

## Abstract

In recent years, greenhouse-grown tomato (*Solanum lycopersicum*) plants showing vascular wilt and yellowing symptoms have been observed between 2015 and 2018 in North Carolina (NC) and considered as an emerging threat to profitability. In total, 38 putative isolates were collected from symptomatic tomatoes in 12 grower greenhouses and characterized to infer pathogenic and genomic diversity, and mating-type (*MAT*) idiomorphs distribution. Morphology and polymerase chain reaction (PCR) markers confirmed that all isolates were *Fusarium oxysporum* f. sp. *lycopersici* (FOL) and most of them were race 3. Virulence analysis on four different tomato cultivars revealed that virulence among isolates, resistance in tomato cultivars, and the interaction between the isolates and cultivars differed significantly (*P* < 0.001). Cultivar ‘Happy Root’ (*I-1, I-2*, and *I-3* genes for resistance) was highly resistant to FOL isolates tested. We sequenced and examined for the presence of 15 pathogenicity genes from different classes (*Fmk1*, *Fow1*, *Ftf1*, *Orx1*, *Pda1*, *PelA*, *PelD, Pep1*, *Pep2*, *eIF-3*, *Rho1*, *Scd1*, *Snf1*, *Ste12*, and *Sge1*), and 14 *Secreted In Xylem* (*SIX*) genes to use as genetic markers to identify and differentiate pathogenic isolates of FOL. Sequence data analysis showed that five pathogenicity genes, *Fmk1, PelA*, *Rho1*, *Sge1*, and *Ste12* were present in all isolates while *Fow1*, *Ftf1*, *Orx1*, *Peda1*, *Pep1*, *eIF-3*, *Scd1*, and *Snf1* genes were dispersed among isolates. Two genes, *Pep2* and *PelD*, were absent in all isolates. Of the 14 *SIX* genes assessed, *SIX1, SIX3, SIX5, SIX6, SIX7, SIX8, SIX12*, and *SIX14* were identified in most isolates while the remaining *SIX* genes varied among isolates. All isolates harbored one of the two mating-type (*MAT-1* or *MAT-2*) idiomorphs, but not both. The *SIX4* gene was present only in race 1 isolates. Diversity assessments based on sequences of the effector SIX3*-* and the translation elongation factor 1-α encoding genes *SIX3* and *tef1-*α, respectively were the most informative to differentiate pathogenic races of FOL and resulted in race 1, forming a monophyletic clade while race 3 comprised multiple clades. Furthermore, phylogeny-based on *SIX3-* and *tef1-*α gene sequences showed that the predominant race 3 from greenhouse production systems significantly overlapped with previously designated race 3 isolates from various regions of the globe.

## Introduction

The soil-borne fungus *Fusarium oxysporum* Schlecht. (anamorph) is a species complex and varies considerably in morphological and pathological characteristics (Booth, 1971; O’Donnell et al., 2009). Members of this species complex infect many important host plants worldwide and typical symptoms are characterized by chlorosis of leaves, necrosis of vascular systems, and even death of colonized plants (Armstrong and Armstrong, 1975; Gordon and Martyn, 1997). Strains are classified into forma specialis (f. sp.) based on specialization to parasitize specific hosts (Armstrong and Armstrong, 1981; Katan, 1999). *Fusarium oxysporum* f. sp. *lycopersici* (FOL) (Sacc.) W. C. Snyder and H. N. Hans is a xylem-colonizing fungus and has long been known to cause wilt on tomato in the United States (Walker, 1971; Nelson et al., 1983) and world-wide. Fusarium wilt of tomato has been commonly managed by planting resistant cultivars in fields (Stall, 1961; Anonymous, 1985; Jones et al., 1991).

The disease symptoms caused by FOL have been seen in multiple greenhouses in North Carolina (NC) before the current study. Based on our surveys, disease incidence ranged from 10 to 90% depending on tomato cultivars and greenhouse sites. Previous studies have documented genetic variations in FOL populations from fields (Volin and Jones, 1982; Davis et al., 1988; Chellemi et al., 1992; Marlatt et al., 1996; Bost, 2001), but pathogenic and genomic diversity of FOL isolates from greenhouses has not been investigated yet.

Traditionally, the identification and classification of pathogenic races of FOL were based on testing pathogenicity on different tomato cultivars that harbor different immunity (*I*) genes to confer resistance (*R*) (Takken and Rep, 2010). Three distinct pathogenic races have been reported worldwide (Armstrong and Armstrong, 1981; Mes et al., 1999). Race 1 is avirulent on tomato genotypes that have the *I-1* gene, race 2 is virulent on *I-1* genotypes but avirulent on *I-2* genotypes, and race 3 is virulent on *I-1* and *I-2* genotypes but avirulent on *I-3* and *I-7* genotypes (Mes et al., 1999; Gonzalez-Cendales et al., 2016). Race 1 (Booth, 1971) and race 2 (Alexander and Tucker, 1945) were first reported in 1886 and 1945, respectively. In 1978, race 3 was detected in Australia (Grattidge and O’Brien, 1982). Subsequently, race 3 was found in several states in the United States (Volin and Jones, 1982; Davis et al., 1988; Chellemi et al., 1992; Marlatt et al., 1996; Bost, 2001), and Mexico (Valenzuela-Ureta et al., 1996). Although cultivar-specific pathogenicity was useful, this test was slow and laborious. To complement the conventional methods, molecular techniques have been used to investigate genetic differentiation, genetic diversity, phylogeny, and classification of pathogenic variants of *Fusarium oxysporum* to host plants.

One subset of genes includes pathogenicity genes. Such genes are directly involved in disease development under natural conditions, but these genes are not necessary to complete the life cycle of a pathogen *in vitro* (Schäfer, 1994; Idnurm and Howlett, 2001). Furthermore, these genes are classified according to their roles in the formation of infection structures, cell wall degradation, suppression of plant immunity, ability to respond to the host environment, production of toxins, and in signal cascades (Idnurm and Howlett, 2001; Jones and Dangl, 2006; van der Does and Rep, 2007; Cross, 2008; Hogenhout et al., 2009).

The term fungal effector refers to any protein synthesized by a pathogen that is exported to a potential host, which has the effect of making the host environment more beneficial to the pathogen (Lo Presti et al., 2015). Pathogenic strains of *Fusarium oxysporum* produce *Secreted In Xylem* (*SIX*) genes, and 14 *SIX* genes have been reported so far (Rep et al., 2004; Houterman et al., 2007; Lievens et al., 2009; Takken and Rep, 2010; Schmidt et al., 2013; Taylor et al., 2016). The products of these genes are small cysteine-rich proteins secreted by FOL in infected-tomato plants (Houterman et al., 2007; van Dam et al., 2016). The molecular markers developed from these genes have provided robust PCR-based methods for identifying the host specificity of FOL isolated from plant tissues (Lievens et al., 2009; Jelinski et al., 2017). For example, the *SIX4* gene was able to identify race 1 isolates while sequence variations in the *SIX3* gene can differentiate race 2 from race 3 isolates of FOL (Lievens et al., 2009). Typically, *SIX1, SIX3*, and *SIX5* can act as avirulence (*AVR*) genes as they are recognized by immune receptors: *I-3*, *I-2*, and *I-1*, respectively (Rep et al., 2004, 2005; Houterman et al., 2008, 2009; Ma et al., 2015). Some evidence suggests that these genes can also act as virulence factors to promote host colonization through the manipulation of the hormone pathways and modulation of plant immunity (Dodds and Rathjen, 2010; de Sain and Rep, 2015; Ma et al., 2015). The presence of individual *SIX* genes and sequence variations within *SIX* genes have been identified and used to discriminate between isolates and races of FOL and several other formae speciales including *betae*, *canariensis*, *cepae*, *ciceris*, *conglutinans*, *cubense*, *fragariae*, *lilii, medicaginis, melonis, niveum, passiflorae*, pisi, *radicis*-*cucumerinu*m, *radicis-lycopersici*, *raphani, vasinfectum* and *zingiberi* (Lievens et al., 2009; Chakrabarti et al., 2011; Meldrum et al., 2012; Covey et al., 2014; Fraser-Smith et al., 2014; Laurence et al., 2015; Taylor et al., 2016; van Dam et al., 2016). Besides, FOL, the *SIX6* gene has been identified in *F. oxysporum* f. sp. *melonis, F. oxysporum* f. sp. *radicis-cucumerinum* and *F. oxysporum* f. sp. *vasinfectum* (Lievens et al., 2009; Chakrabarti et al., 2011), and the *SIX7* gene in *F. oxysporum* f. sp. *lilii* (Lievens et al., 2009). Although most *SIX* genes have been detected in field populations of FOL worldwide (Lievens et al., 2009), there is no or little information on the distribution and diversity of the *SIX* genes in FOL isolates from greenhouses and they may function to discern diversity within local populations.

In filamentous ascomycetes, the mating-type (*MAT*) locus has been cloned (Arie et al., 2000), and dissimilar ‘idiomorphic’ forms were referred to *MAT1-1* and *MAT1-2* genes (Turgeon and Yoder, 2000). Sexual reproduction occurs between individuals of the opposite mating type (Kronstad and Staben, 1997). The reproductive mode and *MAT*-based phylogenetic analyses were used to infer the evolution and genotypic diversity within *Fusarium oxysporum* (Arie et al., 2000; Clay and Kover, 1996) and reported three distinct lineages in FOL possibly occurring due to asexual reproduction (Kawabe et al., 2005). These genes offered information regarding the diversity of FOL greenhouse isolates.

The main goal of the current study was to circumscribe the problem as to the causal agent, differentiate the race(s) responsible, and to identify the presence and diversity of known pathogenicity genes and *SIX* genes in individual isolates. We characterized the 38 isolates of FOL from 12 tomato greenhouse production systems in NC through sequencing of housekeeping, pathogenicity genes, and *SIX* genes and compared the presence of these genes with the ability of the isolates to cause disease in tomato cultivars. We further examined the distribution of each *MAT* gene in all isolates of FOL to infer reproductive modes of FOL isolates. The information gained into race dynamics, genomic diversity, and genes associated with pathogenicity in FOL may inform disease management strategies in greenhouse tomato production systems.

## Materials and Methods

### Collection and Isolation of the Pathogen

**FIGURE 1:**
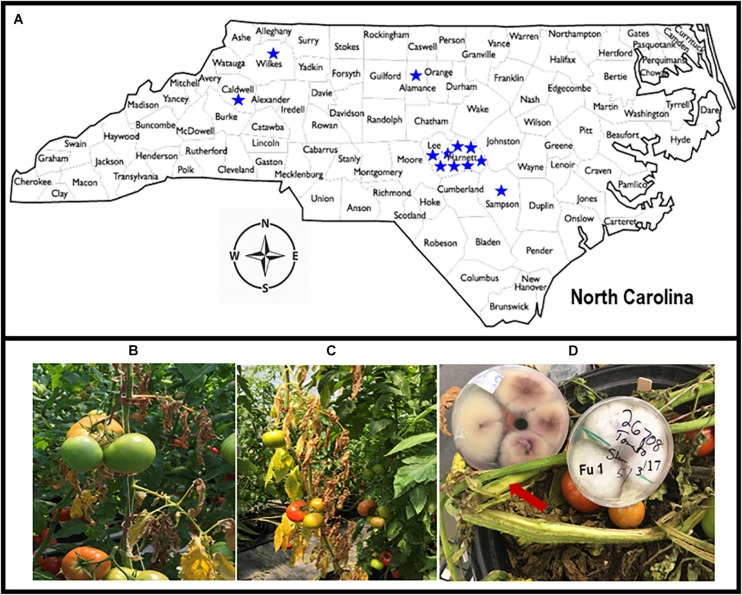
Map of North Carolina showing locations from where diseased tomato plants were collected from 12 greenhouses in six counties **(A)**. Tomato plants showing yellow and wilt symptoms **(B,C)**. The presence of pinkish or brown discoloration or browning of the vascular systems (with arrow) caused by *Fusarium oxysporum* f. sp. *lycopersici* (FOL) on tomato cultivars that were grown in the greenhouses in North Carolina, and creamy and light pink to purple mycelial grown on acidic potato dextrose agar plate containing isolate Fu 1 of FOL **(D)**.

Greenhouse tomato growers with known problems, based on historical data from the Plant Disease and Insect Clinic (PDIC), North Carolina State University (NC State), Raleigh, NC, United States were contacted to ascertain current problems. Site visits were conducted to each greenhouse with cultured tomatoes with putative *Fusarium* wilt symptoms. Symptomatic tomato plants were collected from 12 greenhouses from the piedmont and foothills and eastern in NC in 2018; additional isolates were obtained from the PDIC but originally isolated between 2015 and 2017 (Figure 1A). Tomato plants showing typical wilt and yellowing symptoms (Figures 1B,C) were cut with a sterile razor and the presence of pinkish or brown discoloration of the vascular systems was examined (Figure 1D). To detect the causal agent, four stem sections (∼3 mm^2^) were cut from the symptomatic tomato plants and submerged in 70% ethanol for 30 s and rinsed only one time in sterile distilled water. The stem sections were further disinfested within 3% Clorox (Clorox Company, Oakland, CA, United States) for 1 min and rinsed three times in sterile distilled water before plating. The four surface-sterilized sections were transferred with tweezers into Petri-dishes containing an acidified PDA (A-PDA) (4 g of potato starch, 20 g of dextrose and 15 g of agar/L of distilled water and amended with two antibiotics: ampicillin @ 0.06 g/L and rifampicin @ 0.024 g/L of medium). To identify the fungus, pure cultures incubated for 7 days at 28°C were examined using a dissecting microscope and a total of 38 single-spore isolates were recovered from 12 tomato greenhouses in NC (Table 1; note Fu 8 is not included). For long-term storage, mycelial plugs were prepared from each isolate and stored at −80°C.

**TABLE 1:** Descriptions of the 38 isolates of *Fusarium oxysporum* f. sp. *lycopersici* collected from greenhouse tomato in North Carolina and used in this study.

**Isolate**	**Host cultivar from which isolated**	**Geographic origin (county)**	**Greenhouse Number**	**Year collected**	**PCR-based identification**					**Mating type**	**MtSSU gene^b^**	**Clade^c^**
					**Uni**	**sprl**	**Sp13**	**Sp23**	**Combined PCR assay^a^**			
Fu 1	Geronimo	Harnett	1	2017	+^d^	−^e^	+	−	3	1	−	A
Fu 2	Brandy Boy	Alamance	2	2017	+	−	+	+	1	1	−	B
Fu 3	Trust	Harnett	3	2017	+	−	+	+	3	2	−	C
Fu 4	Primo	Sampson	4	2017	+	−	+	+	3	1	+	D
Fu 5	Better Boy	Lee	5	2015	+	−	+	−	1	1	+	B
Fu 6	Unknown	Caldwell	6	2017	+	−	+	+	3	2	+	n/a^f^
Fu 7	Trolls	Wilkes	7	2015	+	−	+	+	3	1	+	A
Fu 9	Big Dena	Harnett	8	2016	+	−	+	+	3	2	+	C
Fu 10	Trust	Harnett	8	2018	+	−	+	+	3	1	+	A
Fu 11	Trust	Harnett	8	2018	+	−	+	+	3	1	+	C
Fu 12	Geronimo	Harnett	8	2018	+	−	+	+	3	1	+	n/a
Fu 13	Geronimo	Harnett	8	2018	+	−	+	+	3	1	+	A
Fu 14	Geronimo	Harnett	8	2018	+	−	+	+	3	1	+	A
Fu 15	Geronimo	Harnett	8	2018	+	−	+	+	3	1	−	A
Fu 16	Geronimo	Harnett	8	2018	+	−	+	+	3	1	+	A
Fu 17	Geronimo	Harnett	8	2018	+	−	+	+	3	1	+	n/a
Fu 18	Big Dena	Harnett	8	2018	+	−	+	+	3	1	+	A
Fu 19	Big Dena	Harnett	8	2018	+	−	+	+	3	1	−	A
Fu 20	Big Dena	Harnett	8	2018	+	−	+	+	3	1	−	A
Fu 21	Big Dena	Harnett	8	2018	+	−	+	+	3	1	−	A
Fu 22	Big Dena	Harnett	8	2018	+	−	+	+	3	2	−	D
Fu 23	Trust	Harnett	9	2018	+	−	+	+	3	2	+	C
Fu 24	Trust	Harnett	9	2018	+	−	+	+	3	2	+	C
Fu 25	Trust	Harnett	9	2018	+	−	+	+	3	2	−	C
Fu 26	Trust	Harnett	9	2018	+	−	+	+	3	2	+	C
Fu 27	Margureti	Harnett	9	2018	+	−	+	+	3	2	−	C
Fu 28	Margureti	Harnett	9	2018	+	−	+	+	3	2	+	C
Fu 29	Margureti	Harnett	9	2018	+	−	+	+	3	2	−	C
Fu 30	Margureti	Harnett	9	2018	+	−	+	+	3	2	−	C
Fu 31	Trust	Harnett	9	2018	+	−	+	+	3	2	+	C
Fu 32	Unknown	Harnett	10	2018	+	−	+	+	3	2	+	C
Fu 33	Unknown	Harnett	10	2018	+	−	+	+	3	2	−	C
Fu 34	Unknown	Harnett	10	2018	+	−	+	+	3	2	−	D
Fu 35	Unknown	Harnett	10	2018	+	−	+	+	3	2	−	D
Fu 36	Fedrick	Harnett	11	2018	+	−	+	+	3	2	+	C
Fu37	Taymyr	Harnett	11	2018	+	−	+	+	3	2	+	C
Fu 38	Muchoo	Harnett	11	2018	+	−	+	+	3	2	+	C
Fu 39	Sungold	Harnett	12	2018	+	−	+	−	1	1	+	B

*^*a*^Presence of a gene was confirmed by polymerase chain reaction (PCR) assay and sequencing. Uni primers amplified a 670 bp fragment from F. o. f. sp. lycopersici and F. o. f. sp. radicis-lycopersici; sprl primer amplified 947-bp fragment from F. o. f. sp. radicis-lycopersici; Sp13 primers amplified a 445 bp fragment from F. o. f. sp. lycopersici races 1 and 3, and Sp23 primers amplified a 518 bp fragment from F. o. f. sp. lycopersici races 2 and 3 (Hirano and Arie, 2006). ^*b*^Presence of a gene mitochondrial small subunit (mtSSU) by PCR assay and sequencing. ^*c*^Each isolate sequence was analyzed, and clade was determined based on the amplification of the SIX3 effector gene in Figure 6. ^*d*^+ = The presence of each gene was confirmed by PCR assay and sequencing. ^*e*^ - = The absence of each gene was confirmed by PCR assay. ^*f*^n/a = data were not included for these isolates due to short or missing sequences.*

### Identification of the Fungus

Single-spore subcultures were grown on A-PDA at 28°C for 7 days in alternating dark and light 12-h photoperiods. Morphological characteristics such as colony growth, colony texture and pigmentation, the appearance of macro- and micro-conidia, shape, and the number of septa in macro-conidia were examined as described previously (Booth, 1971; Nelson et al., 1983).

To extract genomic DNA, three fungal plugs were transferred to a 250 mL glass flask containing 50 mL of half-strength potato broth (Difco Laboratories, Detroit, MI, United States) for 5 days on a shaker at 100 rpm. Mycelia were harvested by filtration with sterile Mira cloth and then frozen. Approximately 100 mg of mycelia were ground into powder under liquid nitrogen with a mortar and pestle. Genomic DNA was extracted using a DNeasy Plant Maxi Kit (Qiagen, Valencia, CA, United States) according to the manufacturer’s protocols. DNA was quantified using a NanoDrop ND-1000 spectrophotometer (NanoDrop Technologies, Wilmington, DE, United States). Each DNA sample was adjusted to 10 ng/μL using nuclease-free water and stored at 4°C. To identify the fungus, we ran the PCR as described previously (O’Donnell et al., 1998). The sequences of the translation elongation factor 1-α-encoding gene *tef1-*α of the test isolates were compared with the sequences from the National Center for Biotechnology Information (NCBI) database GenBank^1^ as described below.

### Pathological and Molecular Characterization

Pathogenicity is the ability of a pathogen to cause disease on a host (i.e., qualitative property) (Shaner et al., 1992; Casadevall and Pirofski, 1999). ‘Bonny Best’, a universal susceptible cultivar was used to test the pathogenicity of the isolates. Inoculum of each isolate was adjusted to approximately 1 × 10^6^ conidia/mL using a hemocytometer and used to inoculate 3 weeks-old-tomato seedlings. Three seedlings per isolate were inoculated by root-dipping seedlings in a 100 mL inoculum suspension for 15 min. The negative control was treated with sterile distilled water. Inoculated plants were kept on a greenhouse bench and symptom development was monitored until 21 days after inoculation (DAI). The infected stem tissues exhibiting disease symptoms were brought to the laboratory and sections of stem tissues were plated on A-PDA agar plates to isolate the fungus. To complete Koch’s postulates, a hyphal tip portion of each isolate was transferred aseptically and cultured on an A-PDA plate for 7 days. Following sporulation, conidia were harvested and used to re-inoculate 3 weeks-old-tomato seedlings of ‘Bonny Best’ as described above. To further confirm *Fusarium* forma specialis, re-inoculated plants were selected 21 DAI, and crowns were cut with razor blades to check vascular discoloration, and crown and root rot symptoms. These stem tissues were used to re-isolate FOL isolates. Seven-days after incubation, the plates were examined for colony growth and color, and conidia morphology as described above.

**FIGURE 2:**
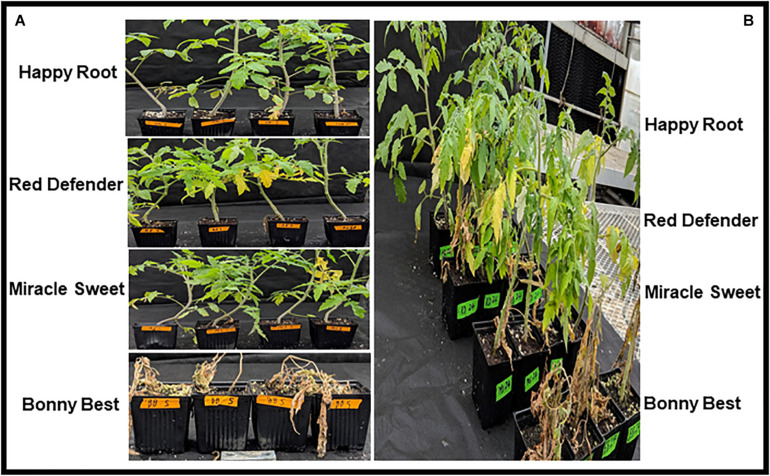
Disease phenotypes caused by *Fusarium oxysporum* f. sp. *lycopersici* on four tomato cultivars 21 days after inoculation in the greenhouse. Tomato differential cultivars inoculated with race 1 isolate Fu 5 **(A)**, and tomato differential cultivars inoculated with race 3 isolate Fu 24 **(B)**.

The term virulence herein is used to describe the capacity of a pathogen to infect a host genotype (i.e., quantitative property) (Shaner et al., 1992; Casadevall and Pirofski, 1999) possessing *I*-genes for resistance to FOL. To identify the race of each isolate of FOL, four differential tomato cultivars were selected and used (Figure 2). The test cultivars included ‘Bonny Best’ (susceptible to races 1, 2, and 3 and used as a susceptible check), ‘Miracle Sweet’ (*I-1* gene for resistance to race 1), ‘Red Defender’ (*I-1* and *I-2* genes for resistance to races 1 and 2), and ‘Happy Root’ (*I-1, I-2*, and *I-3* genes for resistance to races 1, 2, and 3). The seed of the differential cultivars was purchased from Harris Seeds, Rochester, NY. Tomato seedlings were raised in a sandy soil substrate for 2 weeks. The root inoculation method (Williams, 1981) was used. Before inoculation, seedlings were uprooted and washed carefully with tap water to remove excess sand particles. Spore suspensions from seven-day-old cultures (1 × 10^6^ conidia/mL) were used for inoculation. Roots of each cultivar were submerged in the inoculum for 15 min and transplanted to plastic pots containing a sterile 1:1 mixture of sand and soil. Seedlings dipped in sterile distilled water served as negative controls. Twelve seedlings of each cultivar were tested for each isolate and the control. The inoculated seedlings were placed in humidified chambers for 24 h and subsequently grown on a greenhouse bench, where day and night temperatures averaged 36° and 22°C, respectively. The experiments were laid out as a split-split plot design with isolate as the main plot and cultivar as the sub-plot. There were three replications (pots) per isolate and four plants per pot. Disease severity was assessed 7, 14, and 21 DAI as described previously (Marlatt et al., 1996). The area under the disease progress curve (AUDPC) was calculated from disease severities (Shaner and Finney, 1977). Two independent experiments were conducted for virulence analysis. The virulence tests were conducted between October 2018 and August 2019. Data from both experiments were combined and analyzed using a PROC general linear model in SAS v9.4 (SAS Institute, Cary, NC, United States). Differences in virulence among isolates, disease reactions on tomato cultivars, and the interaction between isolates and cultivars were calculated based on AUDPC values and significant differences were estimated at *P* < 0.001 or 0.0001. Virulence of the isolate was determined by the presence or absence and extent of host damage.

For the molecular-based identification and genetic diversity analysis, all primers were synthesized at IDT Inc., Coralville, IA, United States (Supplementary Table S1). *Fusarium* species-specific PCR assays were performed to identify species and forma specialis (Hirano and Arie, 2006). The uni primers were used to confirm *Fusarium oxysporum* while the sprl primers were used to discriminate between *Fusarium oxysporum* f. sp. *lycopersici* (FOL, causes Fusarium wilt) and *Fusarium oxysporum* f. sp. *radicis-lycopersici* (FORL, causes Fusarium crown and root rot). To identify pathogenic races, sp13, and sp23 primers and PCR conditions were used as described previously (Hirano and Arie, 2006). The PCR products were separated on 1.5% (wt/vol) agarose (Applied Biological Materials, Inc., Richmond, BC, Canada) gel in 1 × TAE buffer (40 mM Tris, 20 mM acetic acid, and 1 mM EDTA) containing 0.001% (v/v) Gel red (Biotium, Inc., Union City, CA, United States). A 100 bp DNA marker (Invitrogen™) was loaded in each gel to determine the amplified fragment size. The gel was run under 100 volts for 90 min and photographs were taken using a molecular gel imager (Bio-Rad Laboratories Inc., Hercules, CA, United States).

### Characterization of Genomic Regions Underlying the Pathogenicity Genes and Effector Genes

Fifteen pathogenicity genes involved in signaling pathways, cell-wall degradation, and transcriptional factors regulating gene expression and conferring virulence to *F. oxysporum* (Idnurm and Howlett, 2001; Husaini et al., 2018) and described previously in other *F. oxysporum* f. spp. (Covey et al., 2014; Ellis et al., 2016) were examined. The 15 pathogenicity genes included the Fusarium transcription factor (*Ftf1*), putative oxidoreductase 1 (*Orx1*), pea pathogenicity peptide 1 and 2 (*Pep1)* and (*Pep2*), eukaryotic translation initiation factor 3 (*eIF-3*), ras-like GTP-binding protein (*Rho1)*, stearoyl-CoA desaturase (*Scd1*), carbon catabolite-derepressing protein kinase (*Snf)*, and transcription factor involved in pheromone response (*Ste*12), mitogen-activated protein kinase (*Fmk1*), a mitochondrial carrier protein (*Fow1*), pisatin demethylase 1 (*Pda1*), pectate lyase A (*PelA*), pectate lyase D (*PelD*), and nuclear protein or *SIX* gene expression 1 (*Sge1*) (Supplementary Table S1; Covey et al., 2014; Ellis et al., 2016). The 14 *SIX* genes were assessed to detect the presence of these genes associated with virulence as described previously (Lievens et al., 2009; Meldrum et al., 2012; Covey et al., 2014; Taylor et al., 2016). The confirm the amplification, 5 μL amplified PCR product of each sample was run on a 1.5% (wt/vol) agarose gel stained with Gel red. The remaining 6 μL of each sample was sequenced at the Genomic Sciences Laboratory (GSL), North Carolina State University (NC State), Raleigh, NC, United States.

### Phylogenetic Analysis

To investigate phylogenetic relationships, the mitochondrial small subunit (*mtSSU*), internal transcribed spacer (*ITS*), *tef1-*α, and *SIX3* genes were selected based on our preliminary results and PCR was performed as described previously (White et al., 1990; O’Donnell et al., 1998; Covey et al., 2014). Sequences of each gene were edited by using Geneious v.11.1.4 (1 May 2018, Biomatters Ltd., Auckland, New Zealand). Consensus nucleotide sequences of each gene of each isolate were obtained and used for phylogenetic analysis. Sequences of the FOL representative isolates BFOL-51, and IPO3 and MM10 were included from GenBank as positive controls for race 3 and race 1, respectively, and *F. odoratissimum* isolate Ara1 was used as the outgroup member. The sequences of each gene were analyzed using the Tamura-Nei genetic distance model (Tamura and Nei, 1993). Bootstrap analysis was used to determine the statistical support for each branch of trees generated with 1,000 replications.

### Comparison of the Translation Elongation Factor 1-α Encoding Gene *tef1-*α Sequences Between the Current Isolates From Greenhouse Tomato Production Systems and Field Isolates of FOL

The DNA sequences of the translation elongation factor 1-α-encoding gene *tef1-*α of each isolate of FOL were trimmed and aligned using MAFFT v. 7 (Katoh and Standley, 2013). To further compare the phylogenetic relationships between the isolates from the greenhouses in NC and field populations, *tef1* gene sequences of field isolates of FOL and other *F. oxysporum* f. spp. and *Fusarium* spp. were downloaded from GenBank^2^ (Table 2). Sequences of the field populations of FOL included strains from the United States (isolate MM10 from Arkansas; CA92/95, FOLR2, and DF0-23 from California; BE1, JBF5, MN-24, DA-1, MN-0805, and NRRL 26037 from Florida and OSU451 from Ohio), Australia (isolate 14844), Israel (isolate 24L), The Netherlands (isolate E175) and South Korea (isolate TF103). Also, the *tef1-*α gene encoding the translation elongation factor sequences of 19 other formae speciales of *F. oxysporum* such as *albedinis*, *batatas*, *callistephi*, *cepae*, *cubense*, *dianthi, fabae*, *heliotropii*, *lactucae*, *lini*, *matthiolae*, *medicaginis, melonis*, *narcissi*, *phaseoli*, *radicis-lycopersici*, *rhois*, *spinaciae*, and *vasinfectum*, and four *Fusarium* spp. (e.g., *commune, foetens, hostae*, and *redolens*) were included as outgroup members from GenBank^2^. Phylogenetic relationships were analyzed using the maximum-likelihood (ML) method based on the Jukes-Cantor model (Jukes and Cantor, 1969) with 1,000 replications. Phylogenetic trees were assembled with each isolate of FOL and reference isolates of other *oxysporum* f. spp. and *Fusarium* spp. using the T-BAS v.2.0 (Carbone et al., 2017). The tree was drawn to scale, with branch lengths measured in the number of substitutions per site. A bootstrap value of 80% was considered as the threshold for good confidence.

**TABLE 2:** The geographic location of origins, accession numbers, and isolates of *F. o.* f. sp. *lycopersici* (FOL) sampled from greenhouse tomato production systems in North Carolina (NC) and FOL field and other *Fusarium* spp. isolates in GenBank.

**Species**	**Isolate no.**	**Race**	**State**	**Country**	**Accession no.^a^**
*Fusarium oxysporum* f. sp. *lycopersici*	Fu 1	3	North Carolina	United States	MK917748
isolates from greenhouses in NC	Fu 2	1	North Carolina	United States	MK917749
	Fu 3	3	North Carolina	United States	MK917750
	Fu 4	3	North Carolina	United States	MK917751
	Fu 5	1	North Carolina	United States	MK917752
	Fu 6	3	North Carolina	United States	MK917753
	Fu 7	3	North Carolina	United States	MK917754
	Fu 9	3	North Carolina	United States	MK917755
	Fu 10	3	North Carolina	United States	MK917756
	Fu 11	3	North Carolina	United States	MK917757
	Fu 12	3	North Carolina	United States	MK917758
	Fu 13	3	North Carolina	United States	MK917759
	Fu 14	3	North Carolina	United States	MK917760
	Fu 15	3	North Carolina	United States	MK917761
	Fu 16	3	North Carolina	United States	MK917762
	Fu 17	3	North Carolina	United States	MK917763
	Fu 18	3	North Carolina	United States	MK917764
	Fu 19	3	North Carolina	United States	MK917765
	Fu 20	3	North Carolina	United States	MK917766
	Fu 21	3	North Carolina	United States	MK917767
	Fu 22	3	North Carolina	United States	MK917768
	Fu 23	3	North Carolina	United States	MK917769
	Fu 24	3	North Carolina	United States	MK917770
	Fu 25	3	North Carolina	United States	MK917771
	Fu 26	3	North Carolina	United States	MK917772
	Fu 27	3	North Carolina	United States	MK917773
	Fu 28	3	North Carolina	United States	MK917774
	Fu 29	3	North Carolina	United States	MK917775
	Fu 30	3	North Carolina	United States	MK917776
	Fu 31	3	North Carolina	United States	MK917777
	Fu 32	3	North Carolina	United States	MK917778
	Fu 33	3	North Carolina	United States	MK917779
	Fu 34	3	North Carolina	United States	MK917780
	Fu 35	3	North Carolina	United States	MK917781
	Fu 36	3	North Carolina	United States	MK917782
	Fu 37	3	North Carolina	United States	MK917783
	Fu 38	3	North Carolina	United States	MK917784
	Fu 39	1	North Carolina	United States	MK917785
*F. o.* f. sp. *lycopersici* from fields	MM10	3	Arkansas	United States	FJ790393
	DF0-23	2	California	United States	HM057295
	OSU451	2	Ohio	United States	HM057335
	FOLR2	n/a^b^	California	United States	DQ837692
	CA92/95	3	California	United States	FJ790387
	BE1 (5397)	3	Florida	United States	HM057293
	JBF5	3	Florida	United States	HM057315
	MN-24	3	Florida	United States	HM057331
	DA-1	3	Florida	United States	HM057333
	MN-0805	3	Florida	United States	HM057288
	NRRL 26037	3	Florida	United States	AF008498
	TF103	3		South Korea	KC491844
	FOL-24L	2		Israel	FJ790383
	E175	1		Netherlands	FJ790391
	14844	3		Australia	FJ790386
*F. o*. f. sp. *radicis-lycopersici*	HE-0616		Florida	United States	HM057311
*F. o.* f. sp. *albedinis*	NRRL 26622				DQ837688
*F. o.* f. sp. *batatas*	NRRL 22535				DQ837678
*F. o.* f. sp. *callistephi*	NRRL 25231				DQ837680
*F. o.* f. sp. *cepae*	NRRL 22538				DQ837681
*F. o.* f. sp. *cubense*	E421A3				KP964892
*F. o*. f. sp. *dianthi*	R207				KP964896
*F. o.* f. sp. *fabae*	NRRL 26411				DQ837684
*F. o.* f. sp. *heliotropii*	NRRL 26412				DQ837685
*F. o.* f. sp. *lactucae*	S1				DQ837657
*F. o.* f. sp. *lactucae*	BMP1300				DQ837658
*F. o.* f. sp. *lactucae*	FK09701				DQ837694
*F. o*. f. sp. *lini*	FOLIN				KP964895
*F. o.* f. sp. *matthiolae*	NRRL 22545				DQ837682
*F. o.* f. sp. *medicaginis*	NRRL 22546				DQ837690
*F. o.* f. sp. *melonis*	TX388				DQ837696
*F. o*. f. sp. *narcissi*	FOXN139				KP964902
*F. o.* f. sp. *phaseoli*	NRRL 26445				DQ837686
*F. o.* f. sp. *rhois*	NRRL 26227				DQ837683
*F. o.* f. sp. *spinaciae*	NRRL 26871				DQ837687
*F. o.* f. sp. *vasinfectum*	NRRL 22536				DQ837679
*F. o.* f. sp. *vasinfectum*	FOV14				DQ837695
*F. commune*	NRRL 28387				HM057338
*F. foetens*	NRRL 31852				HM057337
*F. hostae*	NRRL 29889				HM057340
*F. proliferatum*	31X4				DQ837697
*F. redolens*	NRRL 31075				HM057339

*^*a*^Based on the Translation elongation factor 1-α-encoding gene *t***EF***1-*α sequences. ^*b*^ n/a = not available.*

### Mating Type Analysis

To determine the *MAT* gene, genomic DNA from the 38 isolates was amplified with mating-type (MAT) primer combinations. All PCR conditions and cycles were performed (Supplementary Table S1) as described previously (Yun et al., 2000; Ellis et al., 2014). The PCR products were visualized and sized using the 100 bp DNA marker (New England BioLabs Inc., Ipswich, MA, United States).

## Results

### Identification of the Fungus

All isolates exhibited taxonomic features similar to *Fusarium oxysporum* as described previously (Booth, 1971; Nelson et al., 1983). Mycelia were dense, and color and pigmentation of the isolates varied between white to creamy and light pink to purple (Figure 1D). All isolates produced both macroconidia and microconidia. Macroconidia were sickle-shaped but the color, size, and septate of macroconidia varied among isolates. To further confirm the forma specialis, the spr1 primer did not amplify any DNA sample but the uni primers amplified 670 bp fragments from all isolates tested. All 38 isolates were putatively identified as FOL and used for further studies.

### Pathological and Molecular Characterization

**FIGURE 3:**
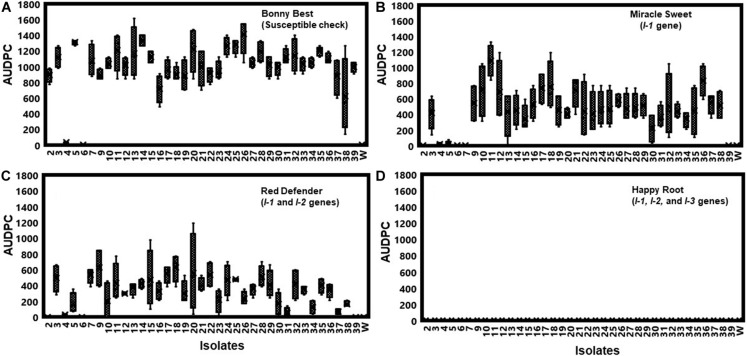
To assess the pathogenicity of each isolate, they were first inoculated on the generally susceptible tomato cultivar ‘Bonny Best’ without the gene for resistance to *Fusarium oxysporum* f. sp. *lycopersici* (FOL). Furthermore, pathogenic races of the 38 isolates of FOL were determined by inoculating them on tomato cultivars possessing different *I*-genes for resistance. Bonny Best (susceptible cultivar) **(A)**. Miracle Sweet (resistant cultivar with *I-1* gene), **(B)**. Red Defender (resistant cultivar with *I-1* and *I-2* genes), **(C)**. Happy Root (resistant cultivar with *I-1*, *I-2* and *I-3* genes), **(D)**. Four tomato cultivars tested were Bonny Best (susceptible check), Miracle Sweet (*I-1* gene), Red Defender (*I-1* and *I-2* genes), and Happy Root (*I-1*, *I-2*, and *I-3* genes) inoculated with each isolate. Three seedlings per isolate were inoculated by root-dipping seedlings in a 100 mL inoculum suspension for 15 min. The negative control was treated with sterile distilled water. Disease severities were recorded 7, 14, and 21 days after inoculation (DAI), and the area under disease progress curve (AUDPC) values were calculated from disease severity ratings. Experiments were conducted twice, and the analysis of results represents combined data. Boxplots and error bars are based on data generated from the full factorial analysis. Race 3 isolate Fu 1 was not included in the analysis due to the few plants affected by water stress. Fu 6 was nonpathogenic isolate. W = water-inoculated plants served as controls.

All isolates (except one isolate: Fu 6) caused typical wilt and chlorosis symptoms on the susceptible cultivar ‘Bonny Best’ (Figure 2) and were like those observed on the original diseased plants. Re-isolated cultures that were inoculated on the susceptible cultivar ‘Bonny Best’ developed typical chlorosis and vascular wilt, thus fulfilling Koch’s postulates. All isolates were nonpathogenic on cv. ‘Happy Root’ (Figure 3). Isolate Fu 4 produced 35, 18, and 26 AUDPC values on ‘Bonny Best’, ‘Miracle Sweet’, and ‘Red Defender’, respectively. Six isolates, Fu 2, Fu 5, Fu 31, Fu 34, Fu 37, and Fu 39 did not induce symptoms or had low AUDPC values on ‘Miracle Sweet’ or ‘Red Defender’. AUDPC values for the remaining isolates ranged from 621 to 1418 on ‘Bonny Best’; 18 to 1085 on ‘Miracle Sweet’, and 53 to 630 on ‘Red Defender’ (Figure 3). Of the 38 isolates assessed, 3 isolates were assigned to race 1, and 34 isolates were race 3 (Table 2). One isolate Fu 6 caused no symptoms in any cultivars tested and was judged to be a nonpathogenic isolate. To further analyze race 3 isolates, ANOVA indicated a significant (*P* < 0.0001) difference in virulence among race 3 isolates and resistance among tomato cultivars, and the interaction between race 3 isolates and cultivars was also significant (*F* value = 3.11 at *P* < 0.0001; Supplementary Table S2). The sp13 primers amplified a DNA fragment of 445 bp from all isolates of race 1 and race 3, while the sp23 primers amplified a DNA fragment of 518 bp from only isolates belonging to race 3. PCR assay confirmed that three isolates (Fu 2, Fu 5, and Fu 39) belonged to race 1 while the remaining 35 isolates were race 3 (Table 1).

### Characterization of Genomic Regions Underlying the Pathogenicity Genes and Effector Genes

**FIGURE 4:**
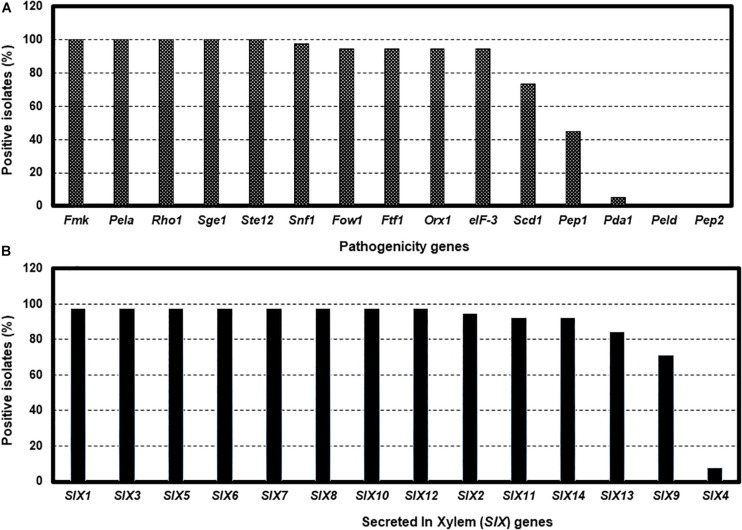
The presence of polymerase chain reaction (PCR) assays targeting the pathogenicity genes and *SIX* genes in the 38 isolates of *Fusarium oxysporum* f. sp. *lycopersici* collected from the 12 tomato greenhouses in North Carolina. The presence was defined as the detection of PCR amplicons and sequences of each gene. The presence of 15 pathogenicity genes **(A)**, and the presence of 14 *SIX* genes **(B)**.

Tests for the presence of the 15 pathogenicity genes by PCR assays revealed considerable variation among isolates. For example, *Fmk1*, *PelA*, *Rhol*, *Sge1*, and *Ste12* genes were present in all isolates, while five genes, *Fow1*, *Ftf1*, *Orx1*, *Snf1*, and *eIF-3* were not detected in several isolates (Figure 4A and Supplementary Table S3). Two genes, *Scd1* and *Pep1* were detected in nearly 74% and 45% of isolates, respectively. The presence (5.3%) of the *Pda1* gene was associated with one nonpathogenic isolate Fu 6 and another pathogenic race 3 isolate Fu 21. In contrast, two genes, *PelD* and *Pep2* were absent in all isolates tested (Figure 4A and Supplementary Table S3). Of the 14 *SIX* genes analyzed, *SIX1, SIX2, SIX3, SIX5, SIX6, SIX7, SIX8, SIX10*, and *SIX12* were detected in more than 95% of the isolates while *SIX9, SIX11, SIX13*, and *SIX14* genes were identified in 70% to 92% of the isolates (Figure 4B). The *SIX4* gene was detected only in three race 1 isolates (Fu 2, Fu 5, and Fu 39) (Figure 4B). In contrast, *SIX5* and *SIX7* genes were absent in the nonpathogenic isolate Fu 6.

### Phylogenetic Analysis

**FIGURE 5:**
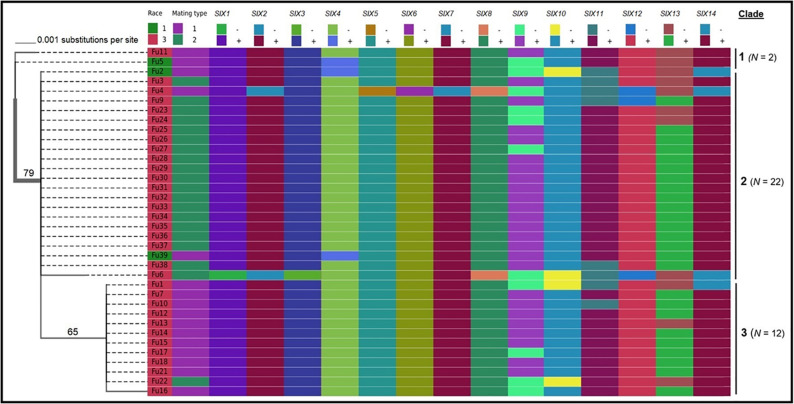
Phylogenetic trees of the isolates of *Fusarium oxysporum* f. sp. *lycopersici* collected from tomato in greenhouses in North Carolina generated from the rDNA internal transcribed spacer region (*ITS*) sequences. The tree topology was obtained through maximum-likelihood with 1,000 replications using the Tamura-Nei DNA substitution model. Parsimony bootstrap values ( > 65%) are shown above the branches. The scale bar indicates 0.001 substitutions per site. Sequences of two isolates Fu 19 and F 20 were not included due to short lengths. For comparisons, pathogenic races, mating types, and the presence or absence of 14 *SIX* genes were included.

**FIGURE 6:**
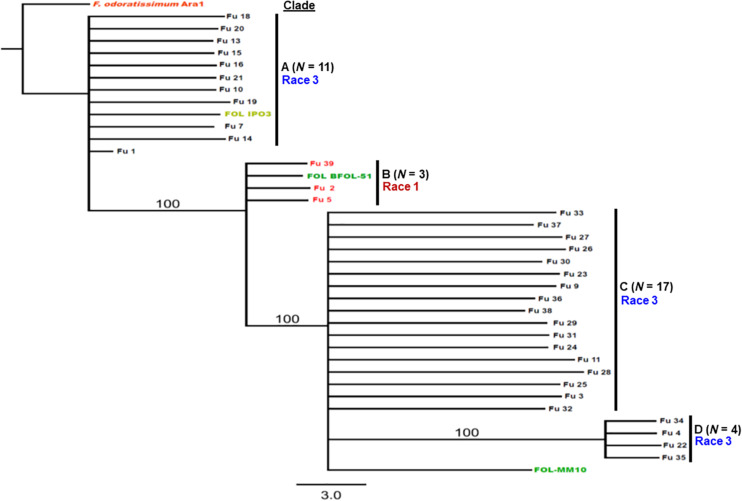
Differentiation of *Fusarium oxysporum* f. sp. *lycopersici* race 3 from race 1 isolates collected from tomato-grown in greenhouses in North Carolina by polymerase chain reaction (PCR) assay targeting the *SIX3* gene and sequence alignments. The Maximum-likelihood phylogenetic trees were generated from the *SIX3* gene sequences of the representative isolates and outgroup isolate “Ara1” of *F. odoratissimum* sequences were downloaded from the National Center for Biotechnology Information database GenBank (http://www.ncbi.nlm.nih.gov/genbank). The tree topology was obtained through maximum-likelihood with 1,000 replications using the Tamura-Nei DNA substitution model. Parsimony bootstrap values ( > 80%) are shown above the branches. The clade of each isolate is also presented in Table 1. Sequences of two isolates Fu 12 and Fu 17 were not included due to short lengths. Nonpathogenic isolate Fu 6 did not have an amplicon corresponding to the *SIX3* gene.

Sequence data analysis showed the *mtSSU* gene product was amplified from nearly 61% of the isolates and these sequences were less informative to discriminate among FOL isolates (Table 1). Although the *ITS* gene region separated the FOL isolates into three clades, this gene was also not useful for distinguishing FOL races due to low taxonomic resolution (Figure 5). The *SIX3* gene-based tree analysis revealed four clades and demonstrated the relationship between the *SIX3* gene and race groupings (Figure 6). For example, clade A had 11 race 3 isolates while clade B contained only three race 1 isolates (Fu 2, Fu 5, and Fu 39) (Figure 6). Clade C consisted of 17 race 3 isolates while clade D contained 4 race 3 isolates and one weakly virulent isolate Fu 4. Within each clade, the *SIX3* gene sequences showed 98 to 100% nucleotide similarity.

### Comparison of the Translation Elongation Factor 1-α Encoding Gene *tef1-*α Sequences Between the Current Isolates From Greenhouse Tomato Production Systems and Field Isolates of FOL

In our initial study, the translation elongation factor 1-α-encoding gene *tef1-*α was successfully amplified from all FOL isolates recovered from greenhouses. The BLAST analysis of the *tef1-*α gene sequence data supported the morphological identification, whereby the closest match (99 -100% similarity) in the NCBI GenBank database was found to be FOL. Furthermore, this locus was chosen because in previous studies this gene revealed the greatest nucleotide diversity when compared with the other loci tested to date within the *F*. *oxysporum* f. spp. complex (O’Donnell et al., 2009; Ellis et al., 2014; Nirmaladevi et al., 2016). The sequences of the *tef1-*α gene were obtained for each isolate and other *F. oxysporum* f. spp. and *Fusarium* species and deposited in the NCBI GenBank database (Table 2).

**FIGURE 7:**
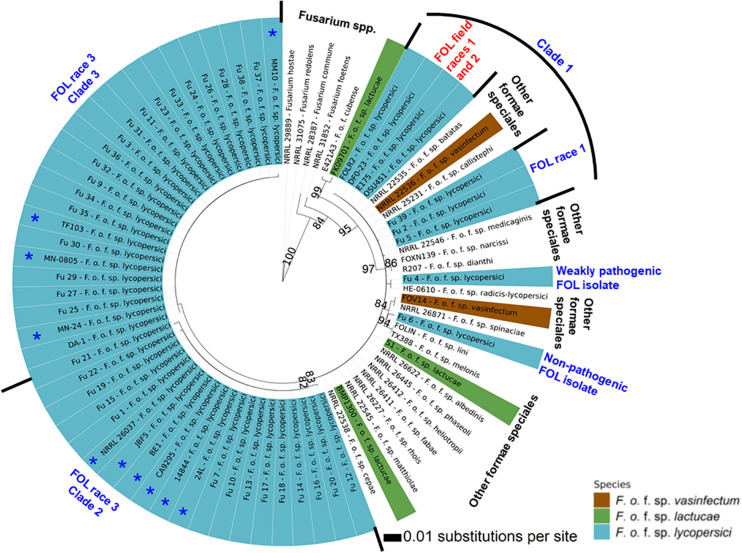
The Maximum-likelihood phylogenetic trees of the 38 isolates of *Fusarium oxysporum* f. sp. *lycopersici* (FOL) sampled from tomato in the greenhouses in North Carolina generated from the translation elongation factor 1-α encoding gene *tef1-* α sequences. For comparison purposes, publicly available the *tef1-*α gene sequences of field isolates of FOL and other formae speciales of *F. oxysporum* and *Fusarium* spp. from the United States and other countries were downloaded from the National Center for Biotechnology Information database GenBank (https://www.ncbi.nlm.nih.gov/genbank/). The tree topology was obtained through maximum-likelihood with 1,000 replications using the Jukes-Cantor model. Phylogenetic trees were assembled with each test isolate using the T-BAS v.2.0 (Carbone et al., 2017). Innermost ring refers to principal component analysis and the numerical values above branches are bootstrap values. Outer ring represents FOL isolates sampled from greenhouses and field isolates from the United States and other countries as well as other formae speciales of *F. oxysporum* and *Fusarium* spp. Asterisk (*) before the isolate number indicates previously reported FOL isolates from fields (Table 2). The isolates joined by dotted lines within the ring indicate these isolates are genetically similar while solid lines represent high nucleotide polymorphisms. Parsimony bootstrap values (>80%) are shown above the branches.

Phylogenetic analysis showed that FOL consisted of at least three major clades. Among them, clade 1 contained three-race 1 isolates from the greenhouses. These isolates were grouped with field race 1 and race 2 isolates and three other *F*. *oxysporum* f. spp. such as *batas*, *callistephi* and *vasinfectum*), which was supported by a bootstrap value of 86% (Figure 7). The nonpathogenic isolate Fu 6 from greenhouse formed a distinct clade with *F. oxysporum* f. spp: *lini*, *melonis*, *spinaciae* and *vasinfectum* with a bootstrap value of 95%. One weakly aggressive ‘race 3’ isolate Fu 4 clustered with *F. o.* f. sp. *radicis-lycopersici*. Interestingly, all 33 FOL isolates recovered from greenhouses and known to be race 3 were clustered into clade 2 and clade 3. Clade 2 contained 14 race 3 isolates from greenhouses in NC along with six race 3 field isolates from Florida (BE1, JBF5, and NRRL 26037), California, (CA92/95), Australia (14844), and Israel (24L). This clade 2 was supported by the bootstrap value of 83% and was 98 to 100% identical to *tef1* gene sequences. ML analysis resulted in clade 3 and all strains appeared nearly identical with a similar bootstrap value of 82% (Figure 7). Clade 3 consisted of 19 race 3 isolates recovered from greenhouses and four previously reported race 3 isolates from fields in Florida (DA-1, MN-24, and MN0805), South Korea (TF103), and from Arkansas (MM10).

### Mating Type Analysis

As expected, a single DNA fragment matching either the *MAT1-1* or *MAT1-2* allele was amplified in each isolate. Based on PCR amplification, a fragment of 593 bp was present for *MAT1-1* and a fragment of 229 bp was amplified for *MAT1-2*. In total, 18 isolates had the *MAT1-1* locus and 20 isolates had the *MAT1-2* locus (Figure 5 and Table 1). The presence of both *MAT* loci was not detected in any isolate tested.

### Pathogenic and Genetic Variation of FOL Isolates Within a Greenhouse

Fu 9 to Fu 22 were isolated from greenhouse # 8 and three different cultivars (Table 1). However, variation existed within this population. The majority were mating type 1 except Fu 9 and Fu 22. Most of the isolates clustered within rDNA clade 3 except Fu 9 and Fu 11 which clustered in clades 2 and 1, respectively (Figure 5). These two isolates also clustered within clade 3 based on the *tef1* gene sequences and all other isolates clustered within clade 3 (Figure 7). Likewise, most isolates were clustered within clade A based on the *SIX3* gene sequence, except Fu 9 and Fu 11 that were in clade C and Fu 22 that clustered in clade D (Figure 6). ANOVA analysis using data from pathogenicity assays on ‘Bonny Best’ only (data not shown) revealed Fu 14 was the most virulent (value of 1330) and was similar to Fu 20, Fu 11, Fu 13, and Fu 15 (AUDPC values in descending order). These were significantly more virulent than the least virulent isolates (Fu 22, Fu 9, Fu 18, and Fu 19, ordered in descending order of AUDPC values) (Supplementary Table S4). The nine isolates from greenhouse #9 all had the same mating type and were present in the same *SIX3* clade and *tef1-*α clade. Fu 26 and Fu 25 were more virulent on ‘Bonny Best’ than Fu 30 and Fu 23; the remaining isolates from greenhouse #9 were intermediate (Supplementary Table S4). The four isolates (Fu 32, Fu 33, Fu 34 and Fu 35) from greenhouse #10 had identical profiles, including virulence on ‘Bonny Best’, but two isolates (Fu 32 and Fu 33) were within the *SIX3* gene clade C and two isolates (Fu 34 and Fu 35) within clade D. The three isolates (Fu 36, Fu 37 and Fu 38) from greenhouse #11 were also identical to one another (mating type 2, *SIX3* gene clade C, *tef1-*α gene clade 3) except Fu 36 were more virulent on ‘Bonny Best’ than Fu 38 while Fu 37 was intermediate (Supplementary Table S4).

## Discussion

Fusarium wilt has not been a major problem in greenhouse tomato production systems in NC due to the deployment of host resistance to known races of FOL. However, multiple reports of wilting and dying greenhouse-tomato plants occurred within a very short and recent period, implying an emerging disease problem, and prompting the need for this study. Isolates were recovered from 12 greenhouses and multiple isolates were cultured, and in some cases, from multiple tomato cultivars, which lack genes for resistance to FOL. The emergent nature of this problem compelled a detailed analysis of the causal agents. All pathogenic isolates were classified as *Fusarium oxysporum* f. sp. *lycopersici*. The data showed a strong cultivar × isolate interaction and mainly races 1 and 3 were present in the greenhouses in NC. Race 1 isolates originated from greenhouses in Alamance, Harnett, and Lee counties while most FOL isolates were race 3 from greenhouses in Harnett, Sampson, and Wilkes counties. To the best of our knowledge, this is the first report of race 3 in greenhouse tomato production systems in NC. Disease assays documented a significant difference in the severity of symptoms and this pathogenic diversity was complemented by assessing the diversity of pathogenicity genes and effector genes within the population.

The 14 *SIX* genes have been identified and investigated (Rep et al., 2004; Houterman et al., 2007; Lievens et al., 2009; Takken and Rep, 2010; Taylor et al., 2016) and their presence or absence, and the gene sequence of *SIX3*, were documented for the isolates in this study. Among these, eight genes (*SIX1, SIX3, SIX5, SIX6, SIX7, SIX8, SIX10*, and *SIX12*) were detected in the majority of the greenhouse isolates while the remaining five genes (*SIX2, SIX9, SIX11, SIX13*, and *SIX14)* were present in some isolates. We found the *SIX4* gene (also known as *AVR1*) only in race 1 isolates and it was absent in all race 3 isolates analyzed (Houterman et al., 2009). *SIX4* or *AVR1* was likely recognized by the *I-1* gene to confer specific resistance in tomato cultivar ‘Miracle Sweet’ to race 1 (Houterman et al., 2008). Two *AVR2* and *AVR3* genes also act as virulence factors in the absence of *I*-genes (Rep et al., 2004; Houterman et al., 2009). Most race 3 isolates identified in this study contained both *SIX3* and *SIX5* or *AVR2* and *SIX1* or *AVR3*, which were recognized by the *I-2*, and *I-2* and *I-3* genes containing cultivar ‘Red Defender’ and ‘Happy Root’, respectively (Rep et al., 2004; Houterman et al., 2009). As a result, cultivar ‘Happy Root’ (*I-1, I-2*, and *I-3* genes) exhibited resistance to both races 1 and 3 isolates of FOL.

The presence of the remaining *SIX* genes in this study has similarly been associated with the pathogenicity of *F. oxysporum* isolates on tomato (*SIX1-SIX7* genes; Lievens et al., 2009), cotton, soybean, common bean (*SIX6* gene; Chakrabarti et al., 2011; Ellis et al., 2016), and banana (*SIX1*, *SIX7*, and *SIX8* genes; Meldrum et al., 2012). *SIX1, SIX3, SIX4, SIX5*, and *SIX6* have all been shown to make a direct contribution to virulence (Rep, 2005; Houterman et al., 2009; Takken and Rep, 2010; Thatcher et al., 2012; Gawehns et al., 2014; Ma et al., 2015). Interestingly, some of these genes can also evade host immunity by suppressing *R* gene-mediated resistance (Jones and Dangl, 2006; Husaini et al., 2018). For example, *SIX1* contributes directly to root penetration and invasion of xylem vessels (Rep et al., 2004, 2005; van der Does et al., 2008; Ma et al., 2010). Remarkably, interactions between FOL *SIX* genes and tomato cultivars with corresponding resistance genes have been useful to distinguish pathogenic races in FOL (Houterman et al., 2008; Takken and Rep, 2010). *SIX8* is a multi-copy gene in FOL but has been detected in formae speciales of cucurbits (van Dam et al., 2016). There are no reports of the function of *SIX7, SIX9, SIX13*, and *SIX14*, awaiting additional research.

Of the 15 pathogenicity genes assessed, five genes (*Fmk1, PelA*, *Rho1*, *Sge1*, and *Ste12*) were present in most isolates, suggesting that these genes may be contributing to pathogenicity of individual isolates of FOL. However, the presence of eight genes (*Fow1*, *Ftf1*, *Orx1*, *Peda1*, *Pep1*, *eIF-3*, *Scd1*, and *Snf1*) showed variation, indicating a partial association of the individual genes with pathogenicity in FOL. These genes also have very close homologs in other *F. oxysporum* f. spp. (Inoue et al., 2002; Martinez-Rocha et al., 2008; Michielse et al., 2009; Rispail and Di Pietro, 2009; Wong Sak Hoi and Dumas, 2010) and have been used to discriminate pathogenic and nonpathogenic isolates, with mixed success (Covey et al., 2014; Ellis et al., 2016). Two genes, *Pep2* and *PelD*, were not amplified in all isolates tested, suggesting that these genes do not have a specific role in pathogenicity on tomato. Our finding showed that one isolate (Fu 6) was nonpathogenic on tomato cultivars but appeared to belong to race 3 using race-specific DNA markers (Hirano and Arie, 2006). Although we were able to detect putative genes that may be contributing to pathogenicity to tomato, we were unable to identify a single genetic marker to differentiate this nonpathogenic isolate from other race 3 isolates of FOL.

The functions of these genes in disease susceptibility have been investigated in the past using molecular, genomic, proteomic, and high-throughput-sequencing approaches. For examples, the *Fmk1* gene was found to be involved in the signal transduction pathway, which can regulate various infection processes of *F. oxysporum* such as the formation of infection hyphae, root attachment and penetration, vascular colonization, and invasive growth on the living plant tissue (Di Pietro et al., 2001). Another gene, *Rho1* was required for structural alterations in the cell walls and virulence (Martinez-Rocha et al., 2008). Target mRNA produced by the *Ftf* gene was tested in *F. o*. f. sp. *phaseoli* and FOL using RNAi gene silencing and attenuation of *Ftf* gene expression resulted in a marked reduction in virulence, indicating that the *Ftf* gene acts as a regulator of virulence of *F. oxysporum* f. spp. (Ramos et al., 2007; Niño-Sánchez et al., 2016; van der Does et al., 2016). *Pda1* encodes a pisatin demethylase that detoxifies the phytoalexin pisatin produced in the roots of pea (Han et al., 2001). *Ste12* encodes a homeodomain transcription factor that regulates invasive growth downstream of the *Fmk1* pathway (Rispail and Di Pietro, 2009). Importantly, *Sge1* can regulate the expression of *SIX* genes, which is required for colonization of the xylem system and disease development (Michielse et al., 2009). Recently, the homolog of the *Sge1* transcription factor has been identified in *F. o.* f. sp. *cubense* TR4, which was involved in colonization of banana roots and pathogenicity (Michielse et al., 2009).

Sequence analysis of the *tef1-*α gene was helpful to understand the diversity of isolates found in NC greenhouses compared to others isolated from various regions of the world. Previous analyses of field populations of FOL identified three races (1, 2, and 3) in the United States including NC (Stall, 1961; Jones and Litrell, 1965; Booth, 1971; Volin and Jones, 1982; Cai et al., 2003). As with the *SIX3* gene analysis, the *tef1-*α gene diversity enabled grouping of race 1 isolates and these were clustered among race 1 and race 2 strains from various regions of the world. Comparative analysis of the *tef1-*α gene sequences between greenhouse isolates and field populations demonstrated that the race 3 clades from greenhouses formed two distinct clades that genetically overlapped with previously identified race 3 field isolates from California and Florida, and even from Australia, Israel, and South Korea. In the United States, FOL race 3 was first reported in Manatee County, Florida (Volin and Jones, 1982) and subsequently, it was disseminated to other tomato-producing states including NC from Florida (Gale et al., 2003). The high level of overlap demonstrates the greenhouse isolates are part of a global population and therefore it is difficult to attribute the source of inoculum to any one source. However, the source of inoculum from an epidemiological perspective is of great importance to the industry. The emergence of the disease, caused predominantly by race 3, indicates introduced inoculum into the greenhouse systems through multiple and independent events. Elucidating the source of this inoculum is an important goal for future studies. Prevention of FOL introductions into a closed greenhouse system is one of the most important integrated disease management (IPM) tactics growers can implement. The *tef1-*α gene sequences also clustered the weakly virulent isolate Fu 4 with *F. o.* f. sp. *radicis-lycopersici* and another nonpathogenic isolate Fu 6 was clustered with the members of other formae speciales such as *F. o.* f. sp. *lini*, *F. o.* f. sp. *melonis*, *F. o.* f. sp. *spinaciae*, and *F. o.* f. sp. *vasinfectum.* These data suggest Fu 4 and Fu 6 are FOL strains.

An important goal in our study was to discern if the emerging problem was due to the same haplotype, suggesting a common source of inoculum. The sequences of the *SIX3* gene were the most informative, partitioning the population into four distinct clades; clade B comprised the race 1 isolates and the race 3 isolates were grouped into three additional clades. These data indicate the isolates do not have a monophyletic origin. In fact, in greenhouse #8 from where multiple isolates were secured, several genotypes were discovered representative of each race 3 clades. These data suggest there were multiple introductions of inoculum. In contrast, isolates from greenhouse #9 were identical to one another, suggesting clonal multiplication of the isolate after an introduction. However, additional work is needed to include larger sample collections from wide geographic regions and more greenhouses to correlate the population structure of the pathogen within each greenhouse with an emerging problem and to validate phylogenetic analysis within races using the *SIX3* and *tef1-*α gene markers.

This study also demonstrated one locus, either *MAT1-1* or *MAT1-2*, but both loci were not found in each isolate. We postulated that the FOL isolates reproduced asexually and had either the *MAT1-1* or *MAT-1-2* gene that was introduced from the fields into the greenhouses. We did not find any direct relationships between the *MAT* genes, or pathogenicity genes or SIX effectors and pathogenic races. Some other *F. oxysporum* spp. carry functional mating-type genes (Arie et al., 2000; Yun et al., 2000). For example, in *F. oxysporum* f. sp. *cubense*, both *MAT* genes were found, and sterile sexual-like structures called ‘perithecia’ were produced (Fourie et al., 2009), indicating that sexual recombination might occur in this fungus (Taylor et al., 1999).

## Conclusion

In conclusion, pathogenic and genomic diversity was robustly documented among FOL isolates recovered from tomato in greenhouses. Both races 1 and 3 were found and race 3 was predominant. The *tef1-*α and *SIX3* genes were useful to investigate the genetic diversity among isolates of FOL. Cultivar ‘Happy Root’ was highly resistant to both races 1 and 3 and cultivars or rootstocks that have the *I-3* gene should confer control of the race 3 problem. Several growers converted to grafted tomato plants once this study identified the emergent problem as FOL race 3 based on known grafting protocols (Louws et al., 2010). Multiple genes associated with pathogenicity and effectors were also characterized within the population and future work on pathogenesis, complemented with research on host genetics, should lead to additional knowledge and methods to limit future emergence of pathogenic variants and losses due to FOL.

## Data Availability Statement

The datasets presented in this study can be found in online repositories. The names of the repository/repositories and accession number(s) can be found below: https://www.ncbi.nlm.nih.gov/genbank/, MK917748-MK918473.

## Author Contributions

TA and FL collected FOL isolates. TA conceived the idea and designed the experiments. AG, TI, and TA performed the experiments, analyzed the data, and prepared the figures. TA and FL wrote the draft. AG, TI, TA, and FL reviewed and revised the manuscript. All authors have read and approved the final manuscript.

## Conflict of Interest

The authors declare that the research was conducted in the absence of any commercial or financial relationships that could be construed as a potential conflict of interest.

## Footnotes

1 https://ncbi.nlm.nih.gov/genbank/

2 https://ncbi.nlm.nih.gov/genbank/
