# Supplementary material for: Pathogenomics Characterization of an Emerging Fungal Pathogen, Fusarium oxysporum f. sp. lycopersici in Greenhouse Tomato Production Systems
Source: Front Microbiol. 2020 Aug 27;11:1995. doi: 10.3389/fmicb.2020.01995 (PMC7482420; doi:10.3389/fmicb.2020.01995)
Supplement: Supplementary file 1 [file Table_1.DOCX]

**Table S1**. Primer sequences and technical polymerase chain reaction (PCR) data used in this study for the identification and

genomic diversity analysis of 38 *Fusarium oxysporum*. sp. *lycopersici* (FOL) isolates collected from 12 greenhouse tomato production systems

|  |  | |  |  |  | |  | |  |  |  |
| --- | --- | --- | --- | --- | --- | --- | --- | --- | --- | --- | --- |
| **PCR method^a^ used** | **Gene** | **Sequence 5'-3' (forward primer/reverse primer)** | | | | **PCR cycles** | | **Annealing temperature (^0^ C)** | | **Amplicon length (bp)^f^** | **Reference** |
| To identify species | *Uni^b^* | ATCATCTTGTGCCAACTTCAG/GTTTGTGATCTTTGAGTTGCCA | | | | 45 | | 54-60 | | 670 | Hirano and Arie, 2006 |
| To identify forma specialis | *Spr1^c^* | GATGGTGGAACGGTATGACC/CCATCACACAAGAACACAGGA | | | | 45 | | 54-60 | | 947 | Hirano and Arie, 2006 |
| To differentiate pathogenic races | *Sp13^d^* | GTCAGTCCATTGGCTCTCTC/TCCTTGACACCATCACAGAG | | | | 45 | | 52-54 | | 445 | Hirano and Arie, 2006 |
| To differentiate pathogenic races | *Sp23^e^* | CCTCTTGTCTTTGTCTCACGA/GCAACAGGTCGTGGGGAAAA | | | | 45 | | 52-54 | | 518 | Hirano and Arie, 2006 |
| To identify *Fusarium* and phylogenetic analysis | *tef1-α* | ATGGGTAAGGAAGACAAGAC/GGAAGTACCAGTGATCATGTT | | | | 35 | | 55 | | 680 | O'Donnell et al.,1998 |
| For phylogenetic analysis | *ITS* | CTTGGTCATTTAGAGGAAGTAA/ TCCTCCGCTTATTGATATGC | | | | 35 | | 59 | | 538 | White et al., 1990 |
| For phylogenetic analysis | *mtSSU* | CAGCAGTCAAGAATATTAGTCAATG/GCGGATTATCGAATTAAATAAC | | | | 35 | | 52 | | 673 | White et al.,1990 |
| To differentiate mating type idiomorphs | *MAT1-1* | GTTTTGACCGCATTGATTGGACTACC/CTACACAACACCCACACTTCTCATCAT | | | | 30 | | 64 | | 593 | Arie et al., 2000 |
| To differentiate mating type idiomorphs | *MAT1-2* | GACCTAGTGCAACAAGAAACAAAAGCGAGTG/GTCACCAGTCGATCAAGGCTCAAC | | | | 30 | | 60 | | 229 | Arie et al., 2000 |
| To detect the gene associated with pathogenicity | *Fmk1* | AGCGACTGCGCATTCTTCTCTACA/TGAGATCACAGTTGGCGTTGAGGA | | | | 35 | | 60 | | 634 | Covey et al., 2014 |
| “ | *Fow1* | ACTATCCGTACCGATCTTCCAGCTCCC/ATGTACCTGAAGGTGTTGCCGACCG | | | | 35 | | 61 | | 742 | Covey et al., 2014 |
| “ | *Ftf-1* | CGTTTCCCTACTAGATCACACCCA/GCTCCTGTATCTCCTCGGAG | | | | 35 | | 60 | | 376 | Covey et al., 2014 |
| “ | *Orx1* | CCAGGCCATCAAGTTACTC/TCTCCAATATGGCAGATTGTG | | | | 35 | | 60 | | 566 | Covey et al., 2014 |
| “ | *Pda1* | CACGAGTGTGGGAGATGAGGAAGGC/AGGCAGAAGTGAGGAGAGCCATAACC | | | | 35 | | 60 | | 569 | Covey et al., 2014 |
| “ | *PelA* | CTGTCAAGGGCAGCTACGATGGTGG/GAGCTGGTGATCTTGCAGGTGTCTCC | | | | 35 | | 60 | | 538 | Covey et al., 2014 |
| “ | *PelD* | GTGAAGTGCTCTGGTCAGGCTGAGG/TGGACTTGTCGCAGCCCTTGTAACC | | | | 35 | | 60 | | -^g^ | Covey et al., 2014 |
| “ | *Pep1* | TGCCTCCAACGCCTCTACAACTATTCC/ACCGTGTGGTTTCTCCTTGACGTGC | | | | 35 | | 60 | | 558 | Covey et al., 2014 |
|  | *Pep2* | ACTCAACATGAAGGTTACGG/CTCCGCGTAGTAATAGTTTCTC | | | | 35 | | 60 | | - | Covey et al., 2014 |
| “ | *eIF-3* | CTAAGTCCACCTCGCCAGATTCC/AGGTAAGGACGGAGTTTGATCGC | | | | 35 | | 60 | | 724 | Covey et al., 2014 |
| “ | *Rho1* | GAAAGCTCGTCATTGTCGGCGATGG/CTCGATGGTCTTCTGGTCGTATCGC | | | | 35 | | 60 | | 747 | Covey et al., 2014 |
| “ | *Scd1* | CTCGGTTTCCAAATTGTCAAGG/CTGGACTCGAGAAGCAATGG | | | | 35 | | 60 | | 376 | Covey et al., 2014 |
| “ | *Sge1* | TCTCTCTGGCAAACTGTTTCATGTCCC/GGTTGTGGTCGGTACTGTTGTTGTGG | | | | 35 | | 60 | | 746 | Covey et al., 2014 |
| “ | *Snf1* | TTTCTCAAGACAAGCTGTGGCAGTCCC/TGTTCTGTTGCTGGGAGGTTCGTCG | | | | 35 | | 61 | | 722 | Covey et al., 2014 |
| “ | *Ste12* | GTGAGATGCCTGTCGTTCCGATTCC/ATCAGAGTGTGGTGAGGCTTCTTCG | | | | 35 | | 61 | | 744 | Covey et al., 2014 |
| To detect the presence of the *SIX* gene associated with virulence | *SIX1* | GTATCCCTCCGGATTTTGAGC/AATAGAGCCTGCAAAGCATG | | | | 35 | | 59 | | 779 | Lievens et al., 2009 |
| “ | *SIX2* | CAACGCCGTTTGAATAAGCA/TCTATCCGCTTTCTTCTCTC | | | | 35 | | 59 | | 749 | Lievens et al., 2009 |
| To differentiate between race 1 and race 3 isolates and phylogenetic analysis | *SIX3* | CCAGCCAGAAGGCCAGTTT/GGCAATTAACCACTCTGCC | | | | 35 | | 59 | | 608 | Lievens et al., 2009 |
| To differentiate between race 1 and race 3 isolates | *SIX4* | TCAGGCTTCACTTAGCATAC/GCCGACCGAAAAACCCTAA | | | | 35 | | 59 | | 967 | Lievens et al., 2009 |
| To detect the presence of the *SIX* gene associated with virulence | *SIX5* | ACACGCTCTACTACTCTTCA/GAAAACCTCAACGCGGCAAA | | | | 35 | | 59 | | 667 | Lievens et al., 2009 |
| “ | *SIX6* | CTCTCCTGAACCATCAACTT/CAAGACCAGGTGTAGGCATT | | | | 35 | | 59 | | 793 | Lievens et al., 2009 |
| “ | *SIX7* | CATCTTTTCGCCGACTTGGT/CTTAGCACCCTTGAGTAACT | | | | 35 | | 59 | | 862 | Lievens et al., 2009 |
| “ | *SIX8* | TCGCCTGCATAACAGGTGCCG/TTGTGTAGAAACTGGACAGTCGATGC | | | | 35 | | 59 | | 250 | Meldrum et al., 2012 |
| “ | *SIX9* | GGGTGGACCATATCACGATGTTCG/GAATACCTGAGTGGAGTTGTGTCTTG | | | | 35 | | 69 | | 458 | Taylor et al., 2016 |
| “ | *SIX10* | GTTAGCAACTGCGAGACACTAGAA/AGCAACTTCCTTCCTCTTACTAGC | | | | 35 | | 65 | | 636 | Taylor et al., 2016 |
| “ | *SIX11* | ATTCCGGCTTCGGGTCTCGTTTAC/GAGAGCCTTTTTGGTTGATTGTAT | | | | 35 | | 61 | | 559 | Taylor et al., 2016 |
| “ | *SIX12* | CTAACGAAGTGAAAAGAAGTCCTC/GCCTCGCTGGCAAGTATTTGTT | | | | 35 | | 61 | | 449 | Taylor et al., 2016 |
| “ | *SIX13* | CCTTCATCATCGACAGTACAACG/ATCAAACCCGTAACTCAGCTCC | | | | 35 | | 61 | | 1027 | Taylor et al., 2016 |
| “ | *SIX14* | ATAAAGTGCGACTGGACTTCTGCC/ACCCCCATCCACATTCCTAAGCGA | | | | 35 | | 67 | | 422 | Taylor et al., 2016 |

|  | *^a^ For each PCR assay, we used a total volume of 25 µL, which contained 2 μL of 10 ng of genomic DNA, 12.5 µL of GoTaq master mix (Promega Corp., Madison, WI), 1 µL each of 10 µM reverse and forward primer, and 8.5 μL of nuclease-free water. All PCR assays were performed using a BioRad PCR thermal cycler* (*Model T100™ Thermal Cycler and iCylcer®, Bio-Rad Laboratories, Inc., Hercules****,*** *CA). PCR conditions for all primers were set at the initial denaturation temperature of 94°C for 5 min, followed by 35 to 45 cycles of 94°C for 1 min, annealing 52 to 69°C for 45 s to 1 min, and elongation at 72°C for 2 min, with an extension at 72°C for 5 to 10 min, and final step 4°C.*  *^b^ Amplified 670 bp fragment from FOL and F. o. f. sp. radicis-lycopersici.*  *^c^ Amplified 947 bp fragment from F. o. f. sp. radicis-lycopersici but not from FOL* |  |
| --- | --- | --- |
|  | *^d^ Amplified 445 bp fragment from FOL races 1 and 3.*  *^e^ Amplified 518 bp fragment from FOL races 2 and 3.*  *^f^ Sequences of each gene were aligned across isolates using Geneious v.11.1.4*.  *^g^ Minus (-) symbol indicates no amplicon detected.* |  |

**Table S2.** Analysis of variance of the area under disease progress curve values based on disease severities in three tomato cultivars inoculated with the 33 isolates of race 3 of *Fusarium oxysporum* f. sp. *lycopersici* in the greenhouse

_____________________________________________________________________________________________________________

**Sources Degree of freedom Sum of square Mean square *F*-value^a^**

_____________________________________________________________________________________________________________

Cultivar^b^ 2 37282646.97 18641323.49 470.26***

Isolate^c^ 32 8737887.35 273058.98 6.89***

Cultivar × Isolate 64 7893781.14 123340.33 3.11***

^_____________________________________________________________________________________________________________________________________________________________________^

^a^ *Cultivars, isolates, and the interaction between cultivars and isolates were significantly different (P < 0.0001).*

*^b^ Three tomato cultivars ‘Bonny Best’ (susceptible to races 1, 2, and 3 and used as a check), ‘Miracle Sweet’ (I-1 gene for resistance to race 1), and ‘Red Defender’ (I-1 and I-2 genes for resistance to races 1 and 2).*

^c^ *Experiments were conducted twice, and the analysis reflect all data from the two experiments*. *Thirty-three isolates of race 3 identified out of 38 isolates of FOL based on virulence on tomato cultivars, and by polymerase chain reaction assay and sequencing.* *Race 3 isolate Fu 1 was not included in analysis due to few plants affected by water stress. Isolate Fu 6 was nonpathogenic on test tomato cultivars (****Figure 3****).*

| **Table S3.** Polymerase chain reaction (PCR) screen targeting the 15 pathogenicity genes in the 38 isolates of *Fusarium oxysporum* f. sp.  *lycopersici* (FOL) collected from 12 greenhouses in North Carolina | | | | | | | | | | | | | | | | | | | | | |
| --- | --- | --- | --- | --- | --- | --- | --- | --- | --- | --- | --- | --- | --- | --- | --- | --- | --- | --- | --- | --- | --- |
| **Isolate** | ***Fmk1*** | ***Fow1*** | ***Ftf1*** | ***Orx1*** | ***Pda1*** | ***PelA*** | ***PelD*** | ***Pep1*** | ***Pep2*** | ***elF-3*** | ***Rho1*** | ***Scd1*** | ***Sge1*** | ***Snf1*** | ***Ste12*** |  |  |  |  |  |  |
| Fu 1 | +^a^ | + | + | + | -^b^ | + | - | + | - | + | + | + | + | + | + |  |  |  |  |  |  |
| Fu 2 | + | + | + | + | - | + | - | - | - | + | + | - | + | + | + |  |  |  |  |  |  |
| Fu 3 | + | + | + | + | - | + | - | - | - | + | + | - | + | + | + |  |  |  |  |  |  |
| Fu 4 | + | + | - | + | - | + | - | - | - | + | + | + | + | + | + |  |  |  |  |  |  |
| Fu 5 | + | + | + | + | - | + | - | - | - | + | + | + | + | + | + |  |  |  |  |  |  |
| Fu 6 | + | + | - | - | + | + | - | + | - | - | + | + | + | + | + |  |  |  |  |  |  |
| Fu 7 | + | + | + | + | - | + | - | + | - | + | + | + | + | + | + |  |  |  |  |  |  |
| Fu 9 | + | + | + | + | - | + | - | - | - | + | + | - | + | + | + |  |  |  |  |  |  |
| Fu 10 | + | + | + | + | - | + | - | + | - | + | + | + | + | + | + |  |  |  |  |  |  |
| Fu 11 | + | + | + | + | - | + | - | - | - | + | + | + | + | + | + |  |  |  |  |  |  |
| Fu 12 | + | + | + | + | - | + | - | + | - | + | + | + | + | + | + |  |  |  |  |  |  |
| Fu 13 | + | + | + | + | - | + | - | + | - | + | + | - | + | + | + |  |  |  |  |  |  |
| Fu 14 | + | + | + | + | - | + | - | + | - | + | + | + | + | + | + |  |  |  |  |  |  |
| Fu 15 | + | + | + | + | - | + | - | + | - | + | + | + | + | + | + |  |  |  |  |  |  |
| Fu 16 | + | + | + | + | - | + | - | + | - | + | + | + | + | + | + |  |  |  |  |  |  |
| Fu 17 | + | + | + | + | - | + | - | + | - | + | + | + | + | + | + |  |  |  |  |  |  |
| Fu 18 | + | + | + | + | - | + | - | + | - | - | + | + | + | + | + |  |  |  |  |  |  |
| Fu 19 | + | + | + | + | - | + | - | + | - | + | + | + | + | + | + |  |  |  |  |  |  |
| Fu 20 | + | + | + | + | - | + | - | + | - | + | + | + | + | + | + |  |  |  |  |  |  |
| Fu 21 | + | + | + | + | + | + | - | + | - | + | + | + | + | + | + |  |  |  |  |  |  |
| Fu 22 | + | + | + | + | - | + | - | + | - | + | + | + | + | + | + |  |  |  |  |  |  |
| Fu 23 | + | + | + | + | - | + | - | - | - | + | + | + | + | + | + |  |  |  |  |  |  |
| Fu 24 | + | + | + | + | - | + | - | - | - | + | + | - | + | + | + |  |  |  |  |  |  |
| Fu 25 | + | + | + | + | - | + | - | - | - | + | + | - | + | + | + |  |  |  |  |  |  |
| Fu 26 | + | + | + | + | - | + | - | - | - | + | + | + | + | + | + |  |  |  |  |  |  |
| Fu 27 | + | + | + | + | - | + | - | - | - | + | + | + | + | + | + |  |  |  |  |  |  |
| Fu 28 | + | + | + | + | - | + | - | - | - | + | + | - | + | + | + |  |  |  |  |  |  |
| Fu 29 | + | + | + | + | - | + | - | - | - | + | + | + | + | + | + |  |  |  |  |  |  |
| Fu 30 | + | + | + | + | - | + | - | - | - | + | + | - | + | + | + |  |  |  |  |  |  |
| Fu 31 | + | + | + | + | - | + | - | - | - | + | + | + | + | + | + |  |  |  |  |  |  |
| Fu 32 | + | + | + | + | - | + | - | - | - | + | + | + | + | + | + |  |  |  |  |  |  |
| Fu 33 | + | + | + | + | - | + | - | - | - | + | + | - | + | + | + |  |  |  |  |  |  |
| Fu 34 | + | - | + | + | - | + | - | + | - | + | + | + | + | + | + |  |  |  |  |  |  |
| Fu 35 | + | - | + | + | - | + | - | + | - | + | + | + | + | - | + |  |  |  |  |  |  |
| Fu 36 | + | + | + | + | - | + | - | - | - | + | + | + | + | + | + |  |  |  |  |  |  |
| Fu 37 | + | + | + | + | - | + | - | - | - | + | + | - | + | + | + |  |  |  |  |  |  |
| Fu 38 | + | + | + | + | - | + | - | - | - | + | + | + | + | + | + |  |  |  |  |  |  |
| Fu 39 | + | + | + | + | - | + | - | - | - | + | + | + | + | + | + |  |  |  |  |  |  |
|  |  |  |  |  |  |  |  |  |  |  |  |  |  |  |  |  |  |  |  |  |  |

*^a^ Pathogenicity gene amplicon was detected in each isolate of FOL based on PCR assays and sequence dataset.*

*^b^ No amplicon was detected based on PCR assays.*
